# CNS Metastases from Bone and Soft Tissue Sarcomas in Children, Adolescents, and Young Adults: Are They Really So Rare?

**DOI:** 10.1155/2017/1456473

**Published:** 2017-01-24

**Authors:** Monika Bekiesinska-Figatowska, Agnieszka Duczkowska, Marek Duczkowski, Hanna Bragoszewska, Anna Romaniuk-Doroszewska, Beata Iwanowska, Sylwia Szkudlinska-Pawlak, Jaroslaw Madzik, Katarzyna Bilska, Anna Raciborska

**Affiliations:** ^1^Department of Diagnostic Imaging, Institute of Mother and Child, Warsaw, Poland; ^2^Clinic of Oncological Surgery of Children and Adolescents, Institute of Mother and Child, Warsaw, Poland

## Abstract

*Purpose*. To check whether primary involvement of brain/spinal cord by bone/soft tissue sarcomas' metastases in children is as rare as described and to present various morphological forms of bone/soft tissue sarcomas' CNS metastases.* Methods*. Patients with first diagnosis in 1999–2014 treated at single center were included with whole course of disease evaluation. Brain/spinal canal magnetic resonance imaging (MRI)/computed tomography were performed in cases suspicious for CNS metastases. Extension from skull/vertebral column metastases was excluded.* Results*. 550 patients were included. MRI revealed CNS metastases in 19 patients (incidence 3.45%), 14 boys, aged 5–22 years. There were 12/250 osteosarcoma cases, 2/200 Ewing's sarcoma, 1/50 chondrosarcoma, 3/49 rhabdomyosarcoma (RMS), and 1/1 malignant mesenchymoma. There were 10 single metastases and 7 cases of multiple ones; in 2 RMS cases only leptomeningeal spread in brain and spinal cord was found. Calcified metastases were found in 3 patients and hemorrhagic in 4. In one RMS patient there were numerous solid, cystic, hemorrhagic lesions and leptomeningeal spread.* Conclusions*. CNS metastases are rare and late in children with bone/soft tissue sarcomas, although in our material more frequent (3.45%) than in other reports (0.7%). Hematogenous spread to brain and hemorrhagic and calcified lesions dominated in osteosarcoma. Ewing sarcoma tended to metastasize to skull bones. Soft tissue sarcomas presented various morphological forms.

## 1. Introduction

Approximately 3% of brain metastases are caused by sarcomas and 1–8% of sarcoma patients are estimated to develop brain metastases [1, 2]. Central nervous system (CNS) involvement in cases of bone and soft tissue sarcomas in children is considered rare. For example, in a big cohort of children with CNS tumors analyzed by Wiens and Hattab, among 1135 patients, there were only 26 with solid nonhematopoietic CNS metastases which account for 2.3% and among those bone and soft tissue tumors constituted not the first but the second most common cause of CNS metastases after kidneys and adrenal glands tumors [3]. These statistics illustrate well the rarity of brain metastases from bone and soft tissue neoplasms in pediatric population. This is an advantageous circumstance as these metastatic lesions carry poor prognosis; the overall mean survival is estimated at 7–16 months, with the majority surviving less than 12 months (e.g., Ewing's sarcoma) and a minority surviving more than 12 months (e.g., osteosarcoma, chondrosarcoma, and rhabdomyosarcoma (RMS)) [4].

In case of quite common metastases to bones, the skull and vertebral column may also be involved and secondarily epidural space, the meninges, and sometimes even brain parenchyma may be involved as a result of the extension from the affected bones. Primary involvement of the brain and spinal cord is much rarer. However many publications are based on the assumption that there are two mechanisms of sarcoma spread to the brain: hematogenous dissemination into the brain parenchyma and contiguous extension from the adjacent bones [5, 6]. Then the frequency of CNS involvement is high, for example, 6.17% in the material of Postovsky et al. [5]. The contiguous extension of skull bones metastases into the intracranial structures and of the vertebral column metastases into the spinal canal is not the subject of this paper. The purpose of this study is to check in the material from a single center whether CNS involvement via hematogenous dissemination into brain parenchyma and spinal canal is as rare as described indeed and to present various morphological forms of bone and soft tissue sarcomas' metastases to the CNS.

## 2. Methods

The reports of brain and spinal canal magnetic resonance imaging (MRI) with use of a 1.5 T scanner or computed tomography (CT) and the archived images (where possible) were retrospectively reviewed in patients with a suspicion of metastatic spread of bone and soft tissue sarcomas to the CNS. Skull and vertebral column metastases were excluded from the study even if the meninges and/or the nervous tissue were secondarily affected. The inclusion criteria were as follows:

1. Diagnosis of a bone or soft tissue sarcoma (non-RMS soft tissue sarcomas and other sarcomas, e.g., clear cell adrenal and renal sarcomas, were not included)
2. First diagnosis between 1999 and 2014

The whole course of the disease was assessed, until 2016.

No ethical approval was required for preparation and publication of this article by institutional Bioethics Committee due to retrospective nature of this study.

## 3. Results

Among 550 patients, 250 had an osteosarcoma, 200 had Ewing sarcoma, 50 had chondrosarcoma, and 50 had soft tissue sarcoma (STS). In the latter subgroup, 49 patients with RMS and one patient with malignant mesenchymoma (MM) were included.

Neuroimaging revealed CNS metastases in 19 patients, 5 girls and 14 boys, aged 5–22 years at the moment of brain metastases diagnosis (MRI in 11 cases, CT in 8) out of 550 reviewed patients' files.

Out of a total number of 250 patients with osteosarcoma, twelve (4.8%) had brain metastases. Out of 200 Ewing sarcoma patients brain metastases were found in 2 (1.0%). We found these metastases in one out of 50 chondrosarcoma patients (2.0%), in 3 patients with RMS, and in one patient with MM (4 out of 50 STS patients = 8.0%). Altogether the incidence of brain metastases in our material was 3.45%.

There were 10 cases of a single metastasis and 7 cases of multiple ones. In 2 RMS cases we dealt only with leptomeningeal spread in the brain and in the spinal cord. Calcified metastases were found in 3 patients and hemorrhagic ones in 4. In one child with RMS there were numerous solid, cystic, and hemorrhagic lesions as well as leptomeningeal spread of the disease.

In the analyzed material 4 patients had brain metastases before the end of treatment—progression during treatment. Among the remaining 15, in 4 brain metastases were considered early relapse (earlier than one year after the end of treatment) and 11 patients had late relapse (median 1.6 years; min 0.2 year, max 6.9 years).

Detailed results are presented in Table 1.

**Figure 1:**
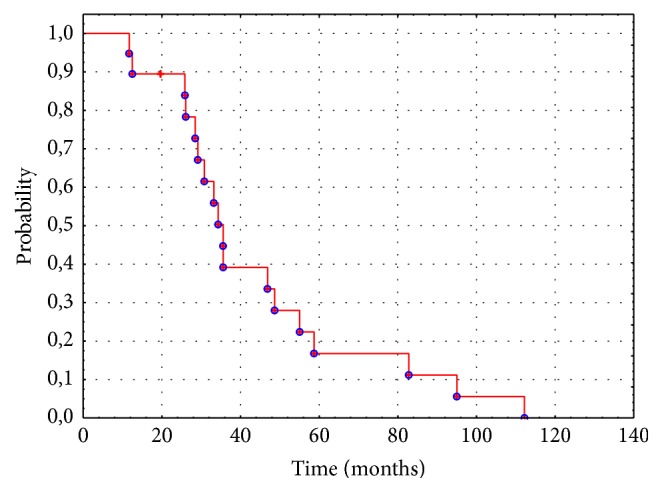
Kaplan-Meier curve of overall survival (OS) for the study group.

In our group of Ewing sarcoma patients with CNS metastatic disease the 5-year overall survival (OS) estimates were 0% and the 3-year OS estimates were 50%. In our osteosarcoma patients with brain metastases the 5-year OS estimates were 18.3% and the 3-year OS estimates were 45.8%. In our patients with RMS and MM with brain metastases the 5-year and 3-year OS estimates were 26%. Our patient with chondrosarcoma has lived one year after the diagnosis of brain metastasis; the 5-year OS estimates were 0% and the 3-year OS estimates were 0%. In the whole analyzed material with a median follow-up of 5 years, the 5-year OS estimates were 16.78% (Figure 1).

## 4. Discussion

Bone and soft tissue sarcomas account for 0.8% of all malignant neoplasms. The development of new chemo- and radiotherapeutic strategies and also surgical resection of lung nodules as a standard of care allow prolongation of survival time through systemic disease control but without effective intracranial control as brain metastases from sarcomas are highly radio- and chemoresistant and surgical resection is the basic treatment. It is also postulated in case of Ewing sarcoma treated by allogeneic stem cell transplantation (SCT) that it may alter typical metastatic patterns “forcing the tumor to escape in unusual sites” [4]. Periodical performing brain imaging is recommended in patients with known active metastatic disease [6]. Particularly active pulmonary metastases are reported as associated with CNS involvement [7, 8] although brain metastases without active disease in the lungs have also been described [9].

The total number of patients with CNS involvement in our material was bigger as there were 2 cases of primary skull tumors (osteosarcoma) as well as 17 cases of metastases to the skull (12 Ewing sarcomas, 2 osteosarcomas, 2 chondrosarcomas, and 1 synovial sarcoma) of which two were accompanied by meningeal and/or parenchymal infiltration. These cases were not included in the study as mentioned above.

**Figure 2:**
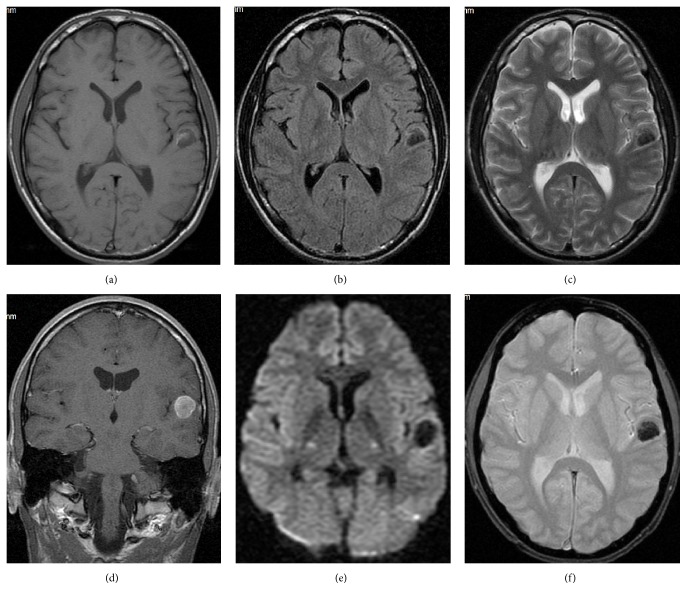
A 16-year-old boy with osteosarcoma of femur. A single calcified metastasis in the left temporal lobe. MRI; SE/T1WI before (a) and after gadolinium-based contrast material injection (d); FLAIR (b); FSE/T2WI (c); DWI (e); GRE/T2*∗*WI (f).

Osteosarcoma is the most frequent malignant bone tumor in children with peak incidence in the second decade of life. Most patients have hematogenous microscopic dissemination at presentation [10] and the most frequent metastatic stations are lungs followed by bones and brain [11]. In case of osteosarcoma, metastasectomy is the standard of care and currently such procedure is an indispensable component of multimodal modern approach focused on better outcome. Osteosarcoma metastases to the brain are described in the literature as in most cases single and located in the frontal lobes [6]. In one-third of our patients (4/12 = 33.3%) there were at least 2 lesions and also in other locations (Figure 2). In our material most of them were solid (in 9/12 cases = 75.0%), not necessarily calcified (in 3/12 cases = 25.0%) and sometimes hemorrhagic (in 4/12 cases = 33.3%).

**Figure 3:**
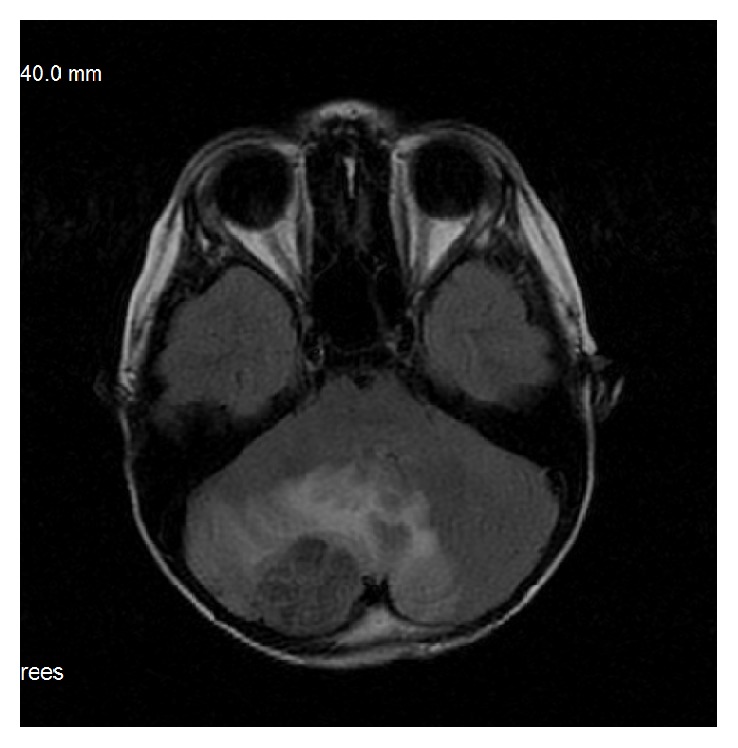
A 9-year-old boy with Ewing sarcoma of the iliac bone. One of three metastases to the brain, in infratentorial location. MRI; FLAIR.

Ewing's sarcoma is the second most frequent malignant bone tumor in children. It is reported as one of the most common sarcomas metastasizing to the brain – up to 56% of cases, but most of them result from the extension of bony metastases into the brain tissue and truly brain metastases make up less than 1.8% of cases [6, 12]. Our cases of truly intraparenchymal metastases resembled meningiomas on MRI with relatively low signal intensity on all sequences and location close to the meninges and—although in the literature reported mainly in supratentorial region—they were also infratentorial (Figure 3) [12].

Although chondrosarcoma is quite a frequent tumor in adults (approximately 30% of all skeletal malignancies), it is uncommon in children. Aprin et al. reported 12 pediatric patients with chondrosarcoma during the period of 23 years with the most frequent location in pelvis, as in our case [13]. Chondrosarcoma metastasizes most frequently to the lungs, less commonly to the soft tissues, lymph nodes, and bone [14, 15]. Brain metastases of chondrosarcoma are extremely rare and only 12 cases are reported in the literature, including two in children [6]. In our material there is only one case of a single metastasis of pelvic chondrosarcoma revealed on CT.

**Figure 4:**
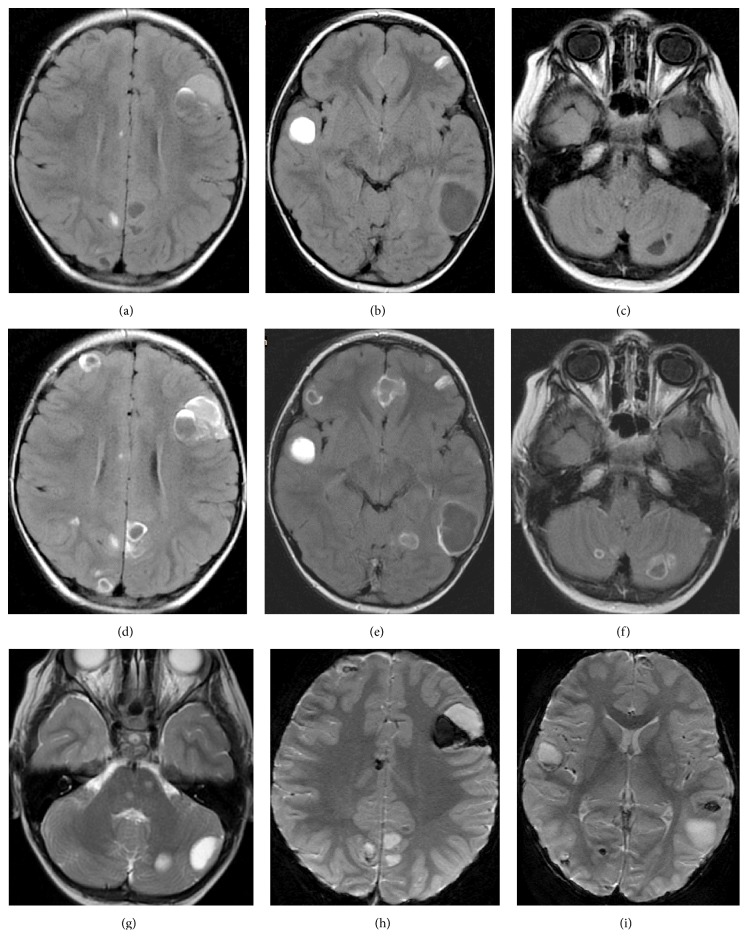
A 5 year-old-boy with foot rhabdomyosarcoma. Enormous number and heterogeneity of metastases form: cystic, hemorrhagic, solid, and leptomeningeal lesions disseminated in both cerebral and cerebellar hemispheres as well in the brain stem. MRI; FLAIR before (a, b, c) and after gadolinium administration (d, e, f); FSE/T2WI (g); GRE/T2*∗*WI (h, i).

**Figure 5:**
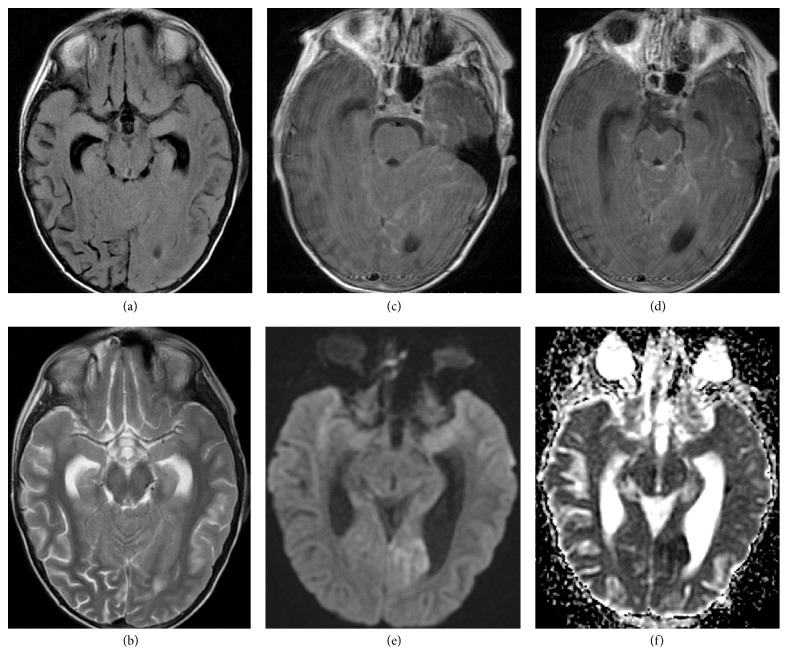
A 7 year-old boy with wrist rhabdomyosarcoma. The metastatic spread is seen only in the leptomeninges. MRI; FLAIR (a); FSE/T2WI (b); SE/T1WI after gadolinium administration (c, d); DWI (e); ADC map (f).

**Figure 6:**
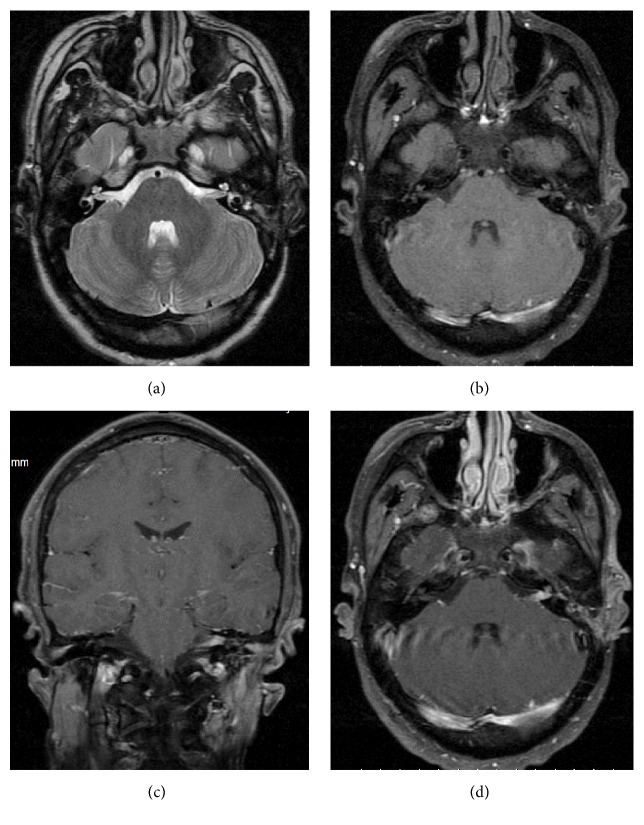
A 25-year-old man with rhabdomyosarcoma of the left cheek. Selective involvement of the left complex of cranial nerves VII and VIII. MRI; FSE/T2WI (a); fat-saturated T1WI before (b) and after gadolinium administration (c, d).

Rhabdomyosarcoma is a common malignant soft tissue tumor in the pediatric population. It is most often located in the head and neck, extremities, and urogenital organs [16] and its metastases are found most frequently in the lungs. It also metastasizes to the bone marrow and bones, omentum, and pleura. The involvement of viscera and brain is rare [17–20]. In our material in one child with RMS there were numerous solid, cystic and hemorrhagic lesions disseminated in both supra- and infratentorial compartment as well as leptomeningeal spread of the disease (Figure 4). In the available literature we found one case of perivascular dissemination in the whole brain of a patient with alveolar RMS of the perimandibular region but reported on pathological examination and not on neuroimaging [7]. In 2 other RMS cases we dealt only with leptomeningeal spread in the brain and in the spinal cord (Figure 5); in one of them only the leptomeninges surrounding the left complex of VII and VIII cranial nerves were involved (Figure 6). In PubMed database we found one earlier report of this type of metastatic spread of RMS in a child [21]. In our two cases the primary tumors were located away from the brain and spinal cord: in the wrist and the foot. However, the last patient—with left nerves VII and VIII involvement—had primary tumor in the left cheek. Arush et al. underline the rapidity of RMS leptomeningeal spread even if the primary tumors are not parameningeal in location. They also stress the low value of normal neuroimaging at diagnosis [21]. This supports the recommendation of performing periodical brain imaging in patients with bone and soft tissue sarcomas [6].

Malignant mesenchymoma is an extremely rare malignant soft tissue tumor composed of two or more different mesenchymal tissue elements (sarcomas) that can occur in any location in the body. Brady et al. reported 8 cases during 22 years; for the last 12 years out of these 22, they have admitted more than 2500 patients with soft tissue sarcomas in one center which illustrates the real rarity of this malignancy [22]. MM metastasizes mainly to the lungs, as all the previously mentioned tumors, and occasionally to the brain [23]. In our material we have one case of a single brain metastasis from pelvic MM with chondrosarcomatous and rhabdomyosarcomatous components.

It is widely accepted in the literature that the time point allowing distinction between early and late relapse is one year after the end of treatment. In the analyzed material in most patients (*n* = 11) brain metastases constituted late relapse (median: 1.6 years after the end of treatment; min 0.2 year, max 6.9 years).

In our study group, 17 out of 19 patients (89.5%) had had lung and/or bone metastases in the earlier phase of the disease, before brain metastases appeared. We therefore believe that intensification of treatment of disseminated disease makes the overall survival (OS) time longer and leaves time for disease dissemination to the CNS. The outcome of bone and soft tissue tumors was much worse in the past, before the era of the combined use of surgery and multidrug intense chemotherapy [24]. It is now well known that, for example, the outcome of Ewing sarcoma patients with isolated lung metastases is similar to that of patients with localized tumor only [25].

In our material the 5-year and 3-year OS estimates for patients with CNS metastatic disease were significantly lower than for patients without this form of spread, as reported in the literature. In a study by Raciborska et al. the 5-year OS estimates for localized Ewing sarcoma were 68.3% and 42% for patients with metastatic disease (non-CNS) [25]. Bielack et al. reported 5-year OS for osteosarcoma patients ranging from 55% to 77%, depending on a center and chemotherapy protocol [24]. In a study by Oberlin et al., 5-year OS for localized RMS was 67%, and in a Weigel study 3-year OS for high risk RMS was 56% [26, 27]. In a Nakamura study the 5-year OS for patients with grade 1 chondrosarcoma was 90%, and only 40%–60% for those with grades 2 and 3 tumors but still higher than in our patient with a brain metastasis [28]. So while isolated lung (or bone) metastases do not affect significantly the overall survival nowadays, the emergence of metastases to the brain significantly reduces survival of bone and soft tissue sarcomas patients.

In these very few papers that describe the incidence of brain metastases, all the solid tumors in children are shown, including neuroblastoma, Wilms' tumor, hepatoblastoma, germ cell tumors, lymphoma, and lung cancer. However, Wiens et al. reported 9 patients out of 526 with bone/soft tissue sarcomas who developed brain metastases which accounts for 1.7% [3]. From the study by Porto et al. one can count the incidence of approximately 0.7% knowing that in their material there were 51 osteosarcomas, 49 Ewing sarcomas, and 48 soft tissue sarcomas, with one case of brain metastasis [29]. Only from the paper of Kebudi et al. we calculated higher incidence of brain metastases (4.3%) as they report them in 12 patients out of 280 children with bone and soft tissue sarcomas that were included in this study group of 1100 patients [30]. With the exception of this last paper, the incidence of brain metastases in our material was higher than in most publications.

## 5. Conclusions

CNS metastases are a rare and late form of spread of the pediatric bone and soft tissues malignant neoplasms, especially osteosarcoma, although in our material they were more frequent (3.45%) than in other, very few reports (most likely due to the fact that we are a big tertiary center managing this specific type of childhood malignancy). Hematogenous spread of the disease to the brain dominated in osteosarcoma as well as hemorrhagic and calcified lesions. Ewing sarcoma tended to metastasize to skull bones. STS metastases presented diverse morphological forms with parenchymal and leptomeningeal involvement.

## Figures and Tables

**Table 1:** 

Number	Gender	Dgn.	Age at first dgn, years	Time from first dgn to CNS mets, years	Number of mets single/multiple	Other mets earlier	Survival after CNS mets, years	Status
1	M	ES	6,9	1,8	Multiple	Lung, bone	0,9	DOD
2	F	ES	12,3	3,8	Single	Lung	2,4	DOD
3	M	OS	14,3	2,7	Single	Lung	1,2	DOD
4	M	OS	11,0	2,5	Multiple	Lung	1,6	DOD
5	M	OS	15,9	3,7	Multiple	Lung	3,2	DOD
6	M	OS	16,5	0,5	Single	Lung, bone	Progression	DOD
7	M	OS	14,9	4,0	Multiple	Bone	3,3	DOD
8	M	OS	12,8	2,1	Single	Lung	0,9	DOD
9	F	OS	9,3	2,2	Multiple	Lung	1,1	DOD
10	M	OS	9,9	1,3	Single	Lung	Progression	Alive
11	M	OS	15,5	1,4	Single	Lung	Progression	DOD
12	F	OS	17,7	3,2	Single	Lung	2,0	DOD
13	F	OS	14,5	6,6	Single	Lung	5,7	DOD
14	M	OS	18,7	9,3	Single	Lung	6,2	DOD
15	M	ChS	12,5	1,9	Single	NA	1,3	DOD
16	M	RMS	12,4	7,5	Pial dissemination	NA	6,9	DOD
17	M	RMS	5,0	0,8	Multiple	Lung, bone	Progression	DOD
18	M	RMS	4,7	1,5	Pial dissemination	Bone	0,2	DOD
19	F	MM	19,9	2,3	Single	Lung	0,7	DOD

M, male; F, female; dgn, diagnosis; CNS, central nervous system; mets, metastases; DOD, died of disease.
